# Increasing prevalence of *Diphyllobothrium* cestodes in seals from the North and Baltic Sea over 26 years

**DOI:** 10.3389/fvets.2025.1574830

**Published:** 2025-08-14

**Authors:** Lotte Caecilia Striewe, Joy Ometere Boyi, Rémi Pigeault, Peter Wohlsein, Ursula Siebert, Kristina Lehnert

**Affiliations:** ^1^Institute for Terrestrial and Aquatic Wildlife Research, University of Veterinary Medicine Hannover, Büsum, Germany; ^2^Department of Pathology, University of Veterinary Medicine Hannover, Hanover, Germany

**Keywords:** zoonotic helminths, harbor seal, grey seal, *Diphyllobothrium schistochilos*, wildlife diseases, aquatic wildlife, environmental change

## Abstract

**Introduction:**

Harbor seals (*Phoca vitulina*) and grey seals (*Halichoerus grypus*) are infected by trophically transmitted intestinal cestodes of the genus *Diphyllobothrium*. *Diphyllobothrium* species can cause zoonotic infections in humans when larval stages are ingested with undercooked fish products. Diphyllobothriid cestode prevalence, infection dynamics, and health impact in phocid seals around densely populated coastal areas are little understood, and their species delineation remains challenging.

**Methods:**

Data collected between 1996 and 2021 within the stranding network of the federal state of Schleswig-Holstein, Germany, were used to analyze cestode prevalence and infection intensity in 1,317 harbor and 153 grey seals from the North Sea and Baltic Sea. A generalized additive model (GAM) assessed host-related factors and longitudinal effects on cestode prevalence in harbor seals from the North Sea (*n* = 1,284). The impact of cestode infections on host health was assessed using histopathological data from intestinal tissue samples. For molecular species identification, cestode DNA was amplified using mitochondrial cytochrome-C-oxidase subunit I (COI) and ribosomal internal-spacer-2 (ITS-2) markers.

**Results and discussion:**

A highly significant increase in cestode prevalence over the 26-year study period was revealed in harbor seals from the North Sea, with prevalences of 0–14% between 1996 and 2012 and 9–36% from 2013 to 2021. Cestode prevalence in grey seals showed significant ecosystem-specific differences and was higher in the Baltic (64%) than in the North Sea (1%). Infection intensities were species-specific, and grey seals exhibited severe infections significantly more often than harbor seals. Histopathological alterations in intestinal tissue were unrelated to cestode infections. Molecular analyses showed that both pinniped species are infected with the same diphyllobothriid species, with the highest sequence similarities of 98.85% (ITS-2) and 90.65% (COI) to *Diphyllobothrium schistochilos*. Increasing cestode prevalence in harbor seals from the North Sea reflects ecosystem changes impacting host–parasite interactions. Clear species- and ecosystem-specific differences are related to differences in immunological traits and ecological conditions, such as the presence of prey species serving as intermediate hosts. Further research on conclusive species identification, health impact, intermediate hosts, and transmission pathways is necessary. The assessment of intermediate hosts and their population dynamics, especially contemplating the impact of environmental change, is crucial for evaluating zoonotic potential and comprehensively assessing the risk for humans.

## Graphical abstract

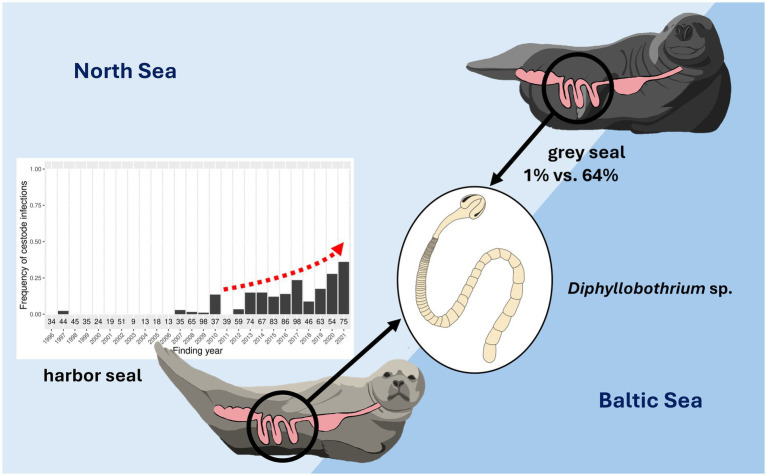

## 1 Introduction

Harbor seals (*Phoca vitulina*) and grey seals (*Halichoerus grypus*) are resident pinniped species in the North Sea and Baltic Sea (1, 2).

After a critical low of harbor and grey seal numbers in the 20th century due to human exploitation, such as unsustainable hunting and pollution, protection measures supported the recovery of both populations, but especially of seals in the Wadden Sea (3, 4). Both seal species exhibit opportunistic, carnivorous feeding behavior (1, 2, 5). As apex predators, they are infected by a variety of trophically transmitted parasites and serve as final hosts for several gastrointestinal helminth species (6, 7). Cestodes of the genus *Diphyllobothrium* infect the intestines of both species (8–10).

In harbor seals, *Diphyllobothrium cordatum*, *Diphyllobothrium elegans*, *Diphyllobothrium hians*, *Diphyllobothrium lanceolatum*, *Diphyllobothrium tetrapterum*, and *Diphyllobothrium schistochilos* are reported, mainly based on morphological identification (11, 12). Taxonomic developments, voucher specimen quality due to age and preservation artefacts, and morphological variability have hampered unequivocal species identification worldwide (11). Cestodes of grey seals were identified as *D hians* in the UK (8). At least *D. cordatum*, *D. elegans, D. hians*, and *D. lanceolatum* are zoonotic and can infect humans when ingested with raw or undercooked fish containing infective larval stages (11).

Diphyllobothriid cestodes have a complex, heteroxenous life cycle including a free-ranging coracidium, two intermediate hosts for the development into pro- and plerocercoids, possible horizontal transfers between intermediate hosts, as well as a definitive (mammal) host to reach maturity (13, 14). Diphyllobothriasis, especially the infection with *Dibothriocephalus latus*, is a human parasitosis and an emerging foodborne zoonosis in the Northern hemisphere (11, 14). Humans are infected by consuming raw or smoked fish products (14). Human infections are facilitated by diphyllobothriids’ low host specificity (14). Among marine species, *Diphyllobothrium stemmacephalum* from toothed whales, for example harbor porpoises, is reported to incidentally infect humans (15). Human infections with other terrestrial species, for example *Dibothriocephalus nihonkaiense* and *Dibothriocephalus dendriticum*, occur frequently, although considered accidental (11, 16). Dietary preferences in Western European countries show a trend towards eating raw fish products, and zoonotic potential is assumed for all diphyllobothriid species (13). Studies on diphyllobothriids in wildlife are scarce (14, 17, 18). Especially marine species are understudied, although two-thirds (37 out of 58) of described diphyllobothriid species are marine, and at least 28 species infect pinnipeds (14). This highlights that more information on species infecting pinnipeds in densely populated coastal areas is needed to evaluate zoonotic and public health risks.

So far, the cestode species infecting pinnipeds in the North Sea and Baltic Sea have have not been identified. Both water bodies, the semi-enclosed North Sea and the “landlocked” Baltic Sea, border Germany and look back at a similar history of anthropogenic use (19–21). However, they differ remarkably in their hydrographic profiles, water qualities, such as salinity and dissolved oxygen, and turnover rates, as well as residence time of introduced matters, such as chemical substances and organic toxins (20). Persistent organic pollutants, as accumulated in the Baltic Sea, are known to affect the health status of marine mammals, e.g., the immune competence towards parasitic infections (22).

Information on infection patterns and pathogenic impact of diphyllobothriids on seal health is scarce. Their epidemiology and changes in prevalence over time need to be monitored, especially in the North Sea and Baltic Sea, where seal populations underwent strong population dynamics. This study investigates *Diphyllobothrium* prevalence and infection trends over a 26-year period, using long-term data from the Schleswig-Holstein stranding network, Germany. Cestodes infecting seals were identified using molecular tools, and infection patterns and health impact in definitive hosts were analyzed.

## 2 Materials and methods

### 2.1 Stranding network, necropsies, and data collection

Within the stranding network in Schleswig-Holstein, Germany, established since 1990, dead or sick marine mammals are reported to local seal rangers. In case of a fatal disease, grey and harbor seals are mercy-killed for animal welfare reasons. Carcasses are transported to the Institute for Terrestrial and Aquatic Wildlife Research (ITAW) in Büsum and examined by experienced veterinarians directly or stored at −20°C and necropsied later. During necropsies, carcasses are classified into degrees of decomposition (ranging from 1 “very fresh” to 5 “mummified carcasses, skeletal remains”) and individual data, including sex (1: male; 2: female) and age class. Age classes are determined as follows: (1) born and deceased in the same year (0–6 months in harbor seals, 0–12 months in grey seals), (2) born in the previous year (up to 18 months in harbor seals and 24 months in grey seals), (3) older than 18 months (harbor seals), older than 24 months (grey seals). For this study, necropsy findings from seal carcasses with a degree of decomposition “very fresh” (1), “fresh” (2), and “well preserved” (3) were considered. In these animals, systematic necropsies are performed, and macroscopic findings are recorded using a standardized protocol established within the stranding network (7, 23, 24). Complete necropsies include opening the intestines and examining systematically over their full length to assess abnormalities and parasitic infections (6). Parasite infections are recorded, level of infection is determined semi-quantitatively as “none” (0), “mild” (1), “moderate” (2), and “severe” (3) during necropsy (7, 23). Associated lesions are recorded, and intestinal sections are preserved in formalin (4%) for subsequent histological investigations. Parasites are collected, rinsed with water, and stored in ethanol (70%) until further identification. For histological examination, tissue samples are fixed in 4% formalin, embedded in paraffin wax, cut into 5 μm sections, and stained with hematoxylin and eosin.

### 2.2 Species, age, and sex distribution of selected seals

Based on the state of decomposition (1–3) and the investigated intestinal tract, 1,317 harbor seals and 153 grey seals, necropsied between 1996 and 2021, were selected for this study (Table 1). Of these, 1,284 harbor seals and 120 grey seals originated from the North Sea, and 33 individuals of each species originated from the Baltic Sea. Sex and age class were noted during necropsy for 1,316 harbor seals and 153 grey seals. Regarding the sexes, the sample size was balanced. Age class distribution was uneven, with a bias towards young animals of age classes 1 and 2. Within the study period, 1,039 seals were shot due to animal welfare reasons, 427 were found dead, and four were reported as bycatch by fishers (Table 1).

**Table 1:** Origin (mercy-killed, found dead, reported bycatch) of examined harbor and grey seals from the North Sea and Baltic Sea.

Seal species	Origin	Mercy-killed	Found dead	Bycaught
Harbor seal (*n* = 1,317)	North Sea (*n* = 1,284)	77% (*n* = 989)	23% (*n* = 293)	>0% (*n* = 2)
	Baltic Sea (*n* = 33)	49% (*n* = 16)	45% (*n* = 15)	6% (*n* = 2)
Grey seal (*n* = 153)	North Sea (*n* = 120)	14% (*n* = 17)	86% (*n* = 103)	0% (*n* = 0)
	Baltic Sea (*n* = 33)	52% (*n* = 17)	48% (*n* = 16)	0% (*n* = 0)

### 2.3 Identification of parasites

**Figure 1:**
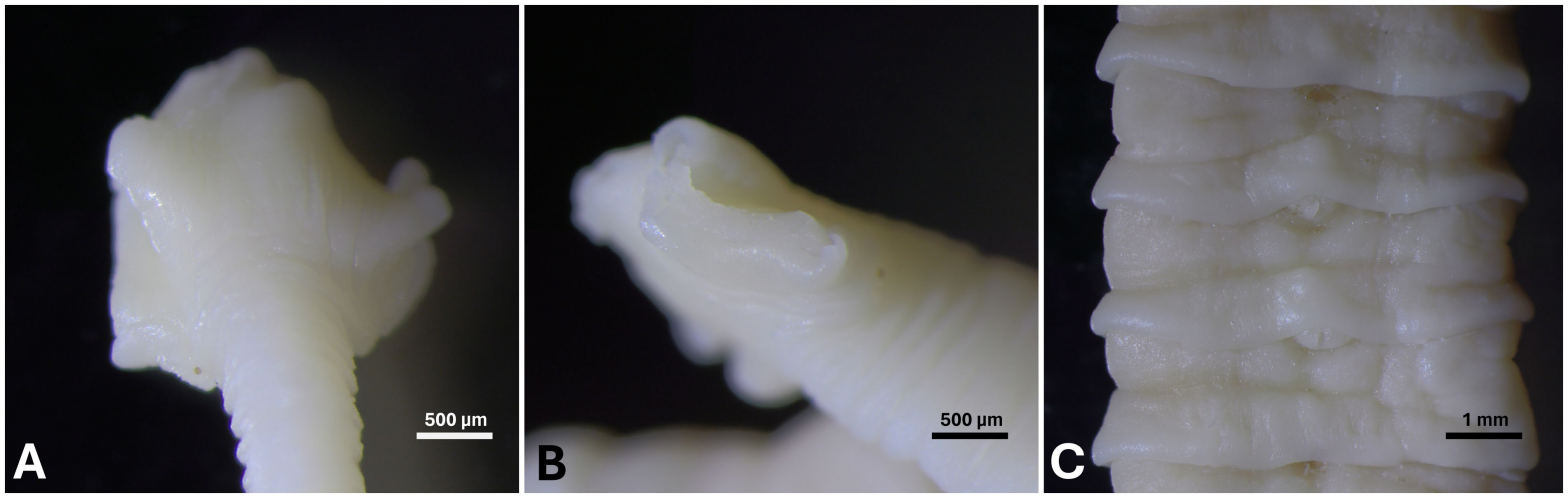
Scolex **(A,B)** and proglottid **(C)** of a *Diphyllobothrium* sp. found in a harbor seal.

Due to their good state of decomposition, 13 cestode specimens were selected for molecular identification. They were collected between 2019 and 2022 from eight harbor seals from the North Sea and four grey seals from the Baltic Sea. Genomic DNA from cestode tissue (proglottids) (Figure 1) was isolated using a QUIamp Micro Kit (Qiagen, Hilden, Germany) according to the manufacturer’s protocol. Voucher specimens were deposited at Senckenberg Institute, Forschungsinstitut und Naturmuseum Frankfurt, Frankfurt, Germany (accession no. SMF 15204 and SMF 15205).

A fragment of mitochondrial DNA (mDNA) of the Cytochrome C oxidase subunit I (COI) was amplified by polymerase chain reaction (PCR) using primer pair JB6 5′-GATAGTAAGGGTGTTGA-3′ and JB5R 5′-CAAGTATCRTGCAAAATATTATCAAG-3′ from Yera et al. (25). Ribosomal DNA of the internal transcribed spacer 2 (ITS-2) was amplified with primer pair ITS2.72F 5′-GCTTTGAACATCGACCTCTTGAAC-3′ and ITS2.692R 5′-ATATGCTTAAGTTCAGCGGGTAATC-3′ designed from *D. cordatum* sequence (accession number: DQ386120.1) published in GenBank using Primer BLAST. PCRs for both primer pairs started with an initial step at 95°C for 1 min, followed by 40 cycles of denaturation (95°C for 15 s), annealing at 53°C (JB6-COI) or 60°C (ITS-2) for 15 s, and elongation (72°C for 10 s). Primer concentrations were 20 pmol/μl, and MyTaq™ Red Mix (BioCat GmbH, Heidelberg, Germany) was used to provide amplification reagents. PCR products that produced a distinct band in electrophoresis were sequenced.

Sequencing reactions were performed at Microsynth Seqlab GmbH (Göttingen, Germany) for each PCR product twice (forward and reverse). Nucleotide sequences were edited and aligned to a consensus sequence using BioEdit (RRID: SCR_007361, Version 7.2.5.0.0). Negative controls without a template were included in PCR reactions.

### 2.4 Statistical analysis

Data analysis was conducted with RStudio (RRID: SCR_000432, Version 4.4.0). For all analyses, the level of significance was set to 0.05.

For time trends in prevalence in mercy-killed and dead-found harbor seals from the North Sea (*n* = 1,284, Supplementary Figure 1), a generalized additive model (GAM) was fitted using the mgcv package (Version: 1.9–1). Presence or absence of cestode infection was fitted as the binomial response variable (1, 0). Year of finding (1996–2021), place of finding (longitude and latitude as a tensor product), sex (male, female), age group (1–3), carcass origin (i.e., mercy-killed or dead-found), and season in which the seal was found were set as explanatory variables. Four seasons were defined as follows, oriented towards the birthing season of harbor seals in summer: 1 (March to May), 2 (June to August), 3 (September to November), and 4 (December to February). Year of finding, origin, age group, and season were tested for interactions before model fitting, but showed only weak interactions. Cubic regression splines were used to assess the effects of the year of finding and the place of finding. The number of knots was set to 4 to limit overfitting. The effects of sex, age group, carcass origin, and season were assessed as random effects. Restricted maximum likelihood (REML) was selected as the smoothness method. Stepwise backward variable selection was used to find the best model fit. Visualization of partial effects for each variable was done using the gratia-package (Version: 0.9.2).

Interspecific and ecosystem-specific differences in cestode prevalence, as well as intraspecific differences in relation to sex and age group, were compared. Significance was evaluated by performing Fisher’s exact or chi-squared test.

Levels of cestode infection were noted in 132 out of 134 infected harbor seals from the North Sea (99%) and all infected grey seals from the Baltic Sea. Performing Fisher’s exact test, the level of cestode infection was compared between host species and age groups.

Results of macroscopic and histological examinations of 79 harbor seals and 16 grey seals infected with cestodes were compared to the results of randomly chosen uninfected harbor seals (*n* = 101) and grey seals (*n* = 8). Presence and absence of diarrhoea (as seen during necropsy), enteritis, and the three most often occurring forms of enteritis were tested against the presence or absence of intestinal cestodes and within the group of infected animals against the level of infection, performing Fisher’s exact test.

## 3 Results

### 3.1 Cestode infections over time

**Figure 2:**
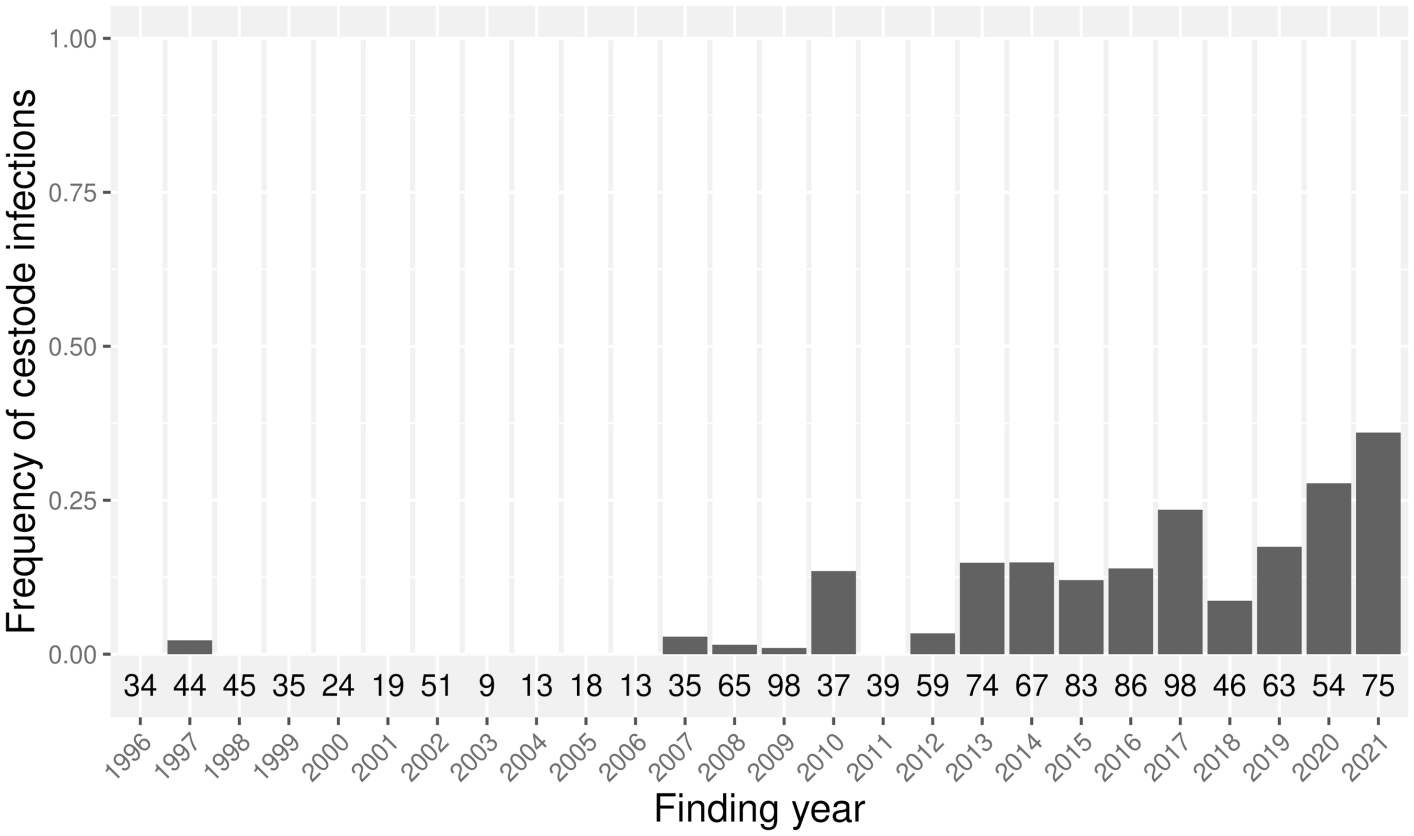
Frequency of cestode infections in harbor seals from the North Sea between 1996 and 2021. The total number of examined harbor seals per year is indicated below each bar.

Cestode infections were found in harbor seals from the North Sea with a yearly prevalence from 0 to 14% until 2012, and 9–36% between 2013 and 2021 (Figure 2). In the Baltic Sea, cestode infections occurred in one harbor seal each in 2016 (50%, *n* = 2) and 2021 (14%, *n* = 7). In grey seals from the North Sea, only one infection occurred in 2021 (20%, *n* = 5). Cestode infections occurred in grey seals from the Baltic Sea in one animal in 2002 (50%, *n* = 2), two animals in 2017 (50%, *n* = 4), and two animals in 2019 (67%, *n* = 3), and in 100% of the individuals examined in 2020 (*n* = 7) and 2021 (*n* = 9).

**Figure 3:**
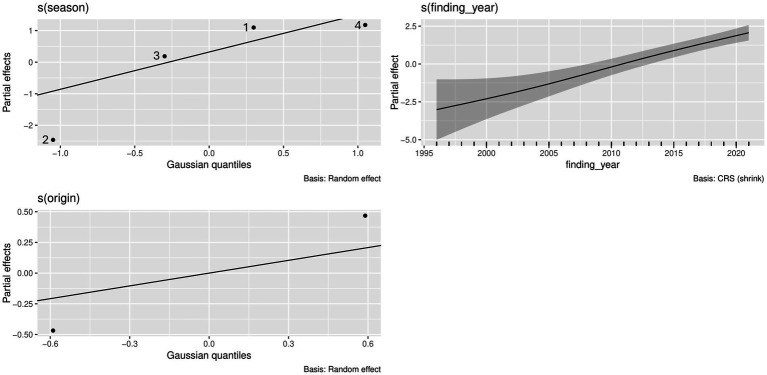
Effects of year of finding as well as season and carcass origin on the presence of cestodes in North Sea harbor seals, based on the applied GAM, and visualized by the draw()-function in the gratia package in R.

In harbor seals from the North Sea, the best model accounted for season, year of finding, and carcass origin, which were all significant (*p* < 0.05) (Table 2 and Figure 3). Prevalence of cestode infections increased over time (*p* < 0.05) and showed significant seasonality, with the highest partial effects in seasons 4 and 1 (*p* < 0.05). The origin of the carcass (mercy-killed or dead-found) had a significant effect on the presence of cestode infection (*p* < 0.05). The model explained 28% of the deviance.

**Table 2:** Significance of the variables used in a generalized additive model (GAM) to assess the presence/absence of cestodes in harbor seals from the North Sea.

Harbor seals from the North Sea				
Independent variable	edf	Ref.df	*χ* ^2^	*p*-value
Season	2.894	3	59.06	<0.05
Year of finding	1.609	3	233.35	<0.05
Carcass origin	0.926	1	28.59	>0.1

*p*-values are estimated by the Wald test, integrated in the mgcv R package.

### 3.2 Ecosystem- and species-specific differences in cestode prevalence

Between 1996 and 2021, 10% of investigated harbor seals (*n* = 1,317) and 14% of investigated grey seals (*n* = 153) were infected with cestodes. Cestode prevalence in harbor seals did not differ significantly between the North Sea (10%) and the Baltic Sea (6%) (Fisher’s exact test, *p* > 0.5). Cestode prevalence in grey seals from the Baltic Sea (64%) was significantly higher than in grey seals from the North Sea (1%) (Fisher’s exact test, *p* < 0.05). Cestode prevalence differed significantly between harbor and grey seal hosts in the North Sea and Baltic Sea (Fisher’s exact test, *p* < 0.05 each) (Table 3).

**Table 3:** Cestode prevalence in grey and harbor seals from the North Sea and Baltic Sea.

Seal species	North Sea	Baltic Sea
Harbor seal	10% (*n* = 1,284)	6% (*n* = 33)
Grey seal	1% (*n* = 120)	64% (*n* = 33)

Within mercy-killed harbor seals from the North Sea (*n* = 989), cestode prevalence was 12%, differing significantly from dead-found harbor seals from the same ecosystem (*n* = 293), for which cestode prevalence was 6% (Fisher’s exact test, *p* < 0.05).

### 3.3 Sex- and age-specific differences in cestode prevalence

Cestode prevalence differed significantly between the age classes in harbor seals from the North Sea: Prevalence in age class 2 was significantly higher (20%) than in age class 1 (8%) or 3 (3%) (Fisher’s exact test, *p* < 0.05).

Cestode prevalence showed no age class-specific differences in grey seals from the Baltic Sea and harbor seals from the North Sea and Baltic Sea (Fisher’s exact test, *p* > 0.1 each). Cestode prevalence did not differ significantly between male and female seals, neither in harbor seals (chi-squared test, *p* > 0.1) nor grey seals (chi-squared test, *p* > 0.1).

### 3.4 Intensities of infection

**Figure 4:**
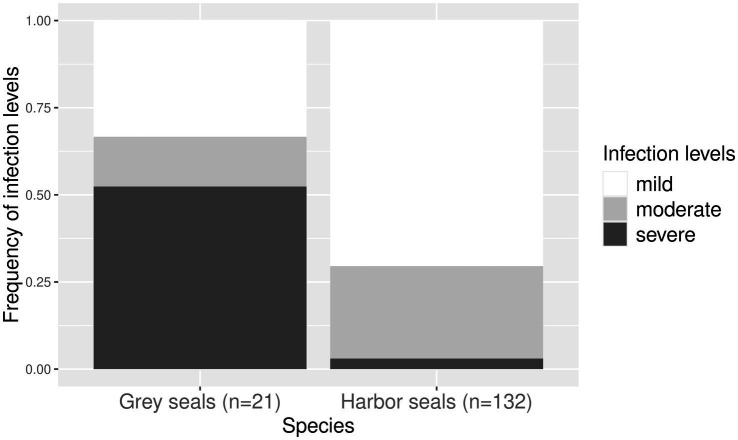
Frequency of infection levels within harbor seals (from the North Sea) and grey seals (from the Baltic Sea) infected with cestodes. Frequency of mild infections is indicated in white, moderate infections in light grey, and severe infections in dark grey.

Infection levels of harbor seals from the North Sea and grey seals from the Baltic Sea within the three age classes are shown in Figure 4. They differed significantly between the two host species (*p* < 0.05). Seventy percent (*n* = 93) of infected harbor seals from the North Sea were mildly infected, 27% (*n* = 35) were moderately infected, and 3% were severely infected (*n* = 4). Fifty-two percent (*n* = 11) of Baltic grey seals were severely infected, 33% were mildly (*n* = 7), and 14% moderately (*n* = 3) infected. No relationship between age class and level of infection was found in harbor seals from the North Sea (*p* > 0.1) and grey seals from the Baltic Sea (*p* = 1).

### 3.5 Pathogenic impact of cestode infection

No significant relations between the presence of cestodes or level of infection and diarrhoea or enteritis were shown in both host species (*p* = 1 each, Table 4).

**Table 4:** Prevalence of diarrhoea and enteritis in harbor seals and grey seals infected with cestodes.

Seal species	Harbor seal			Grey seal		
Pathological finding	(*n* = 79)			(*n* = 16)		
Cestode infection level	Mild (*n* = 53)	Moderate (*n* = 23)	Severe (*n* = 3)	Mild (*n* = 6)	Moderate (*n* = 1)	Severe (*n* = 9)
Diarrhoea	57% (*n* = 30)	57% (*n* = 13)	67% (*n* = 2)	17% (*n* = 1)	100% (*n* = 1)	56% (*n* = 5)
Enteritis	91% (*n* = 48)	87% (*n* = 20)	67% (*n* = 2)	83% (*n* = 5)	100% (*n* = 1)	89% (*n* = 8)

The three most frequent characteristics of intestinal inflammation in harbor seals were eosinophilic (71%), granulomatous (58%), and catarrhal (31%) (Table 5). In grey seals, eosinophilic enteritis occurred most often (50%), followed by catarrhal (63%), and granulomatous and ulcerating inflammation (17% each) (Table 5). No significant relation between the presence/absence of cestode infection or the level of infection and the occurrence of the three most frequently occurring enteritis forms was found in harbor or grey seals (*p* = 1 each).

**Table 5:** Prevalence of the different enteritis forms diagnosed in the two host species; marked in bold are the three most frequent types of enteritis in harbor and grey seals^1^.

Character of inflammation	Harbor seal	Grey seal
	(*n* = 180)	(*n* = 24)
Eosinophilic	**71%**	**50%**
Granulomatous	**58%**	**17%**
Catarrhal	**31%**	**63%**
Supperative-necrotizing	3%	0%
Ulcerating	1%	**17%**
Necrotizing	1%	4%
Erosive	0%	8%

^1^The most frequent inflammatory alterations are marked in bold for each species.

### 3.6 Species identification of cestodes

Good quality ITS-2 forward and reverse sequences (*n* = 20) from eight cestode specimens of eight harbor seal individuals from the North Sea and four cestode specimens of four grey seal individuals from the Baltic Sea were edited and aligned. The derived ITS-2 sequences showed >99% identity with each other. A consensus sequence of 522 bp was blasted in GenBank and showed the highest similarity to *D. schistochilos*, with 98.85% (accession numbers: KY552877.1 and MW601833.1), followed by *D. latus* with identities of 96.55–96.93%.

Good quality cyclooxygenase-1 (COX1) forward and reverse sequences (*n* = 10) from four cestode specimens of four harbor seal individuals from the North Sea and one cestode specimen of one grey seal individual from the Baltic Sea were edited and aligned. The derived COX1 sequences showed >99% identity with each other. A consensus sequence of 674 bp was blasted in GenBank and showed the highest similarity to *D. schistochilos*, with 90.65% (accession numbers: KY552877.1, MW602528.1), followed by *D. tetrapterum* with identities of 89.32–89.47%.

## 4 Discussion

For the first time, the infection dynamics of potentially zoonotic intestinal helminths and an increasing trend in prevalence over a 26-year period in two seal species inhabiting a heavily anthropogenically used and densely populated coastal area are reported. Cestode species infecting harbor and grey seals in German waters were characterized molecularly using two gene loci for the first time and showed strong similarities with *D. schistochilos*. With the results, a valuable insight into species- and ecosystem-specific cestode infections in harbor seals and grey seals in the German North Sea and Baltic Sea is gained.

### 4.1 *Diphyllobothrium* sp. in harbor seals from the North Sea

*Diphyllobothrium* sp. prevalence in harbor seals was 10% in the North Sea during a 25-year study period, with yearly prevalences ranging between 0 and 34%, and a striking increase over the last decade. A prevalence of 31.8% in the German Wadden Sea (10) and 8.5% in the Dutch Wadden Sea (9) was previously reported in harbor seals that died during the phocine distemper virus (PDV) epidemic in 1988. Between 1997 and 2000, no cestode infections were reported in 107 examined harbor seals from the German Wadden Sea (7). In accordance, the majority of cestode infections within the present investigation occurred since 2013 in both the North Sea and the Baltic Sea. In harbor seals from the North Sea, a significant and strong increase in prevalence over time was shown by the GAM.

Harbor seals, as definite hosts and apex predators, are at the top of the *Diphyllobothrium* life cycle cascade in the North Sea. Due to anthropogenic impact, their population size in the North Sea was at a historical low, estimated to be less than 4,000 individuals in the mid-20th century (26). Species as well as habitat protection measures succeeded in a steady population increase, with setbacks due to two PDV epidemics (27–29). Since 2002, the population has grown steadily, although with reduced growth rates in the last years (4, 30). The recovery of harbor seals and their role as definitive hosts for trophically transmitted parasites might have an impact on commercially important fish. However, Diphyllobothriidae have rapid growth and development rates, as well as an extremely high biotic potential (13, 31). They are long-lived, both as plerocercoids in fish and as adults in definitive hosts (13) and show highly successful transmission mechanisms (31). Thus, if a suspected positive influence of the harbor seal recovery on *Diphyllobothrium* sp. populations was unifactorial, the *Diphyllobothrium* sp. increase should have happened much earlier. Regarding their complex life cycle, numerous factors contribute to changing occurrences in definitive hosts. For the human-infecting *Dibothriocephalus latus*, studies suggest that abiotic factors, such as water temperature, water oxygen, and eutrophic level, play key roles in the presence of the cestode in intermediate hosts (32, 33) as well as for the maintenance or prevention of fish infections (33). In optimal environmental conditions, the plerocercoids of *Dib. latus* can survive in fish for months to several years (18, 31), making fish important reservoirs (13). Further, changes in fish abundances might influence the dietary habits of piscivorous fish species and promote or prevent horizontal transfer opportunities for plerocercoids (33, 34). As a shelf sea, the North Sea predominantly consists of coastal and shallow areas (21) and is highly susceptible to human pressures, such as fishing, shipping, and water quality changes (35–37). Furthermore, increasing water temperature, attributed to climate change, has led to a decrease in primary production and a decline in small copepods and fish recruitment over the last decades (38). This “bottom-up effect” and the temperature-induced distribution changes of fish populations (39) could directly impact parasite communities, but also the access of definitive hosts to their prey. Sufficient knowledge on the occurrence of diphyllobothriid species as well as their intermediate and transport hosts is lacking, and causes for increased prevalence in harbor seals are multifactorial.

*Diphyllobothrium* sp. prevalence in harbor seals from the North Sea or the level of infection were not correlated to histopathological changes. In humans, common symptoms of Diphyllobothriasis include diarrhoea and abdominal pain (13, 31). Prolonged infections are associated with low Vitamin B12 levels, and subsequent anemia, massive infections may cause intestinal obstructions (13). Apart from diarrhoea, which was diagnosed in a third of the examined harbor seals, these symptoms are difficult to detect in post-mortem investigations. Future studies focusing on a direct comparison between infected and non-infected parts of the intestine might be able to detect small-scale histopathological changes. Examining biomarkers linked to coproscopic analyses in live individuals could give additional information on how the presence of cestodes impacts live animals.

Apart from the strong increase of *Diphyllobothrium* sp. prevalence in harbor seals from the North Sea in the last 10 years, significant effects of season and origin were detected by the GAM. Additionally, analyses showed that young seals assigned to age class 2 were significantly more often infected than those assigned to age classes 1 or 3. Seasonality of cestode infections, as well as a higher occurrence in young animals, is probably due to life history aspects of parasites and hosts.

In definitive hosts of diphyllobothriids from freshwater environments, seasonal patterns have already been reported. In river otters (*Lontra canadensis*) and wolves (*Canis lupus*) those patterns are ascribed to distinct seasonal access to prey items that serve as intermediate hosts (40, 41). However, wolves and river otters are not strictly piscivorous and have access to prey items not carrying fish tapeworms. *Dib. latus* in intermediate hosts in Italian lakes has the highest prevalence in perch (*Perca fluviatilis*) during autumn and winter, which is attributed to favorable environmental conditions for transmission during the summer (33). In contrast, no seasonal patterns in char (*Salvelinus alpinus*) in Sweden (42), or Baltic three-spined sticklebacks (*Gasterosteus aculeatus*) (43) were detected, potentially due to the longevity of diphyllobothriids. Seasonal differences in access to prey species, and thus intermediate hosts, might explain seasonal variation in *Diphyllobothrium* sp. occurrence in harbor seals from the North Sea. In addition, seasonal reproduction and molting in harbor seals (1) influence dietary habits and immune status. Life history traits were also reflected by age group-specific differences in *Diphyllobothrium* sp. prevalence, but not infection intensity, observed in this study.

In contrast to our findings, a previous study on harbor seals in the German Wadden Sea after a PDV epidemic showed higher *Diphyllobothrium* sp. prevalences and infection intensities with increasing host age (10), suggesting parasite accumulation in one host over its life span. However, that study reflected a snapshot perspective after an epidemic event. Therefore, comparisons to the long-term data presented here are limited.

The GAM showed the strongest presence of cestode infections in seasons 4 and 1 (December to May) for harbor seals from the North Sea, which is the time during which seals of age classes 1 and 2 are in the majority recovered from the coast (44). With the peak of the pupping season of harbor seals from the North Sea being in June (45), and most individuals being weaned by the end of July, *Diphyllobothrium* sp. infections occur predominantly in young seals with a still not fully developed immune competence and a high susceptibility to infection from prey items, as was already proposed for lung nematode infections by Lehnert et al. (7).

Additionally, cestode presence was significantly affected by carcass origin, namely, if an animal was mercy-killed or found dead. As with the strong seasonal effect discussed above, we hypothesize an interplay of age class and origin, as most mercy-killed animals were young, while age class distribution within dead-found animals was more even.

### 4.2 Differences in *Diphyllobothrium* sp. infection patterns of harbor seals and grey seals

This study is the first to report *Diphyllobothrium* sp. infections in grey seals from the German North Sea and Baltic Sea. Grey seals from the Baltic Sea showed a significantly higher *Diphyllobothrium* sp. prevalence (64%) than those from the North Sea (1%). These differences may be due to differing qualities of the two ecosystems (20), reflecting that multistage trophic transmission in intestinal helminths is heavily dependent on environmental conditions, such as salinity, affecting development and occurrence of vertebrate and invertebrate intermediate host species. Surprisingly, harbor seals had similar cestode infection prevalence in both study areas. Further, harbor and grey seals showed significant differences in infection intensities, and grey seals were often severely infected with cestodes. As both host species harbor the same *Diphyllobothrium* species (see below), these species-specific findings might show long-term, if not evolutionary, adaptation processes and immune traits. Harbor seals might be the final host and well-adapted to this *Diphyllobothrium* species.

Although lesions were not associated with cestode infections *per se*, the strong difference between both host species highlights marked differences in their immune responses. This is supported by other pathogen infections whose impacts vary between the two seal species. Grey seals, for example, are rarely affected by lung nematode or PDV infections, which are a major cause of disease in harbor seals in the North Sea (7, 22). It is assumed that harbor seals and grey seals are both suitable hosts for but differently adapted to cestodes of the genus *Diphyllobothrium* sp.

### 4.3 Molecular identification of *Diphyllobothrium* sp.

Both seal species in both ecosystems were infected with the same diphyllobothriid species, belonging to the genus *Diphyllobothrium.* Interestingly, from harbor porpoises (*Phocoena phocoena*) in the German Seas, another diphyllobothriid species, *D. stemmacephalum*, is reported (23, 46). *D. stemmacephalum* also occurs in other cetaceans, like Atlantic white-sided dolphins (*Leucopleurus acutus*) and common bottlenose dolphins (*Tursiops truncatus*) (16). At least in the marine environment, the commonly proposed low host specificity for diphyllobothriids (13) might only apply to animals in the same family. Human infection with *D. stemmacephalum* is possible (15), zoonotic potential of the *Diphyllobothrium* species infecting harbor and grey seals cannot be ruled out. For gene fragments obtained from both primers, BLAST search showed the highest identity with *D. schistochilos*. *D. schistochilos* is reported to occur in different seal species inhabiting Arctic waters, including harbor seals and ringed seals (*Pusa hispida*) (12). However, for the COI marker, sequence identity is lower than 91%. Considering COI is part of the mitochondrial genome, we conclude that the specimens found are most likely a species not yet deposited in GenBank, which is closely related to *D. schistochilos*. In case the specimens are identified as *D. schistochilos*, the intraspecific genetic variation of this species is particularly high. Both scenarios highlight the challenge of diphyllobothriid species identification.

## 5 Conclusion

A strong increase in cestode prevalence in harbor seals over the 26-year study period and ecosystem-specific differences in infection patterns between grey seals from the Baltic Sea and the North Sea were observed. Harbor and grey seals are infected with the same cestode species but show differences in infection intensities and in aspects of intestinal health, indicating species-specific immunological competencies. Time trends in cestode prevalence as well as ecosystem-specific differences reflect the complex life history of heteroxenous parasites. Environmental change may accelerate host–parasite interactions and ecosystem traits influence life cycle viability. Increased *Diphyllobothrium* prevalence in the North Sea emphasizes the One-Health implications and presence of infectious stages in intermediate fish hosts. Their zoonotic potential for infection of humans via consumption of fish in the North and Baltic Sea areas needs to be elucidated further.

## Data Availability

The original contributions presented in the study are included in the article/Supplementary material, further inquiries can be directed to the corresponding author.
